# Controllable Synthesis of Biocompatible Fluorescent Carbon Dots From Cellulose Hydrogel for the Specific Detection of Hg^2+^

**DOI:** 10.3389/fbioe.2021.617097

**Published:** 2021-01-28

**Authors:** Hailong Huang, Hao Ge, Zhipeng Ren, Zhijian Huang, Min Xu, Xianghui Wang

**Affiliations:** School of Physics and Electronic Science & Shanghai Key Laboratory of Magnetic Resonance, East China Normal University, Shanghai, China

**Keywords:** carbon dots, cellulose hydrogel, Hg^2+^ detection, fluorescent sensor, biocompatibility

## Abstract

Heavy metal ions overload can seriously harm human health. Simple and effective strategies for the specific detection of heavy metal ions are of great important. In this work, using different pretreatment methods, a series of carbon dots (CDs) with different particle sizes and doped with varying amounts of elements (O, N, S) were prepared based on the natural polymer, cellulose hydrogel. The CDs exhibit excellent fluorescence and biocompatibility. When the particle size decreased from 8.72 to 2.11 nm, the fluorescence quantum yield increased from 0.029 to 0.183. In addition, doping with elements (N) also effectively enhanced the fluorescent performance of the CDs. The fluorescence of the CDs, especially for the smallest, CD-4a, was significantly quenched in the presence of the heavy metal ion, Hg^2+^. Thus, CD-4a may be used as a fluorescence sensor for the detection of Hg^2+^. The fluorescence intensity of CD-4a exhibited a two-stage, concentration-dependent fluorescence response in the range 0.2–10 and 10–100 μmol/L Hg^2+^, with each stage having different slopes; the detection limit was 0.2 μM. More importantly, even in the presence of interfering metal ions, the detection of Hg^2+^ using the CDs-4a remained stable. Therefore, these biocompatible CDs may serve as a promising candidate for the specific detection of Hg^2+^.

## Introduction

With the development of industrialization, the pollution of the environment with heavy metal ions has become a serious concern all over the world. Heavy metal ions not only harm living organisms in water, but also affect the health of humans (Lu et al., 2018; Bolisetty et al., 2019; Sarma et al., 2019; Wu et al., 2019). The overloading of heavy metal ions such as Cu^2+^, Fe^3+^, Pb^2+^, and Hg^2+^ is highly toxic to biological organisms and causes irreversible, dangerous oxidative stress and serious damage to the central nervous system leading to renal and neural problems (Ali and Khan, 2018; Kahlon et al., 2018; Cai et al., 2019; Kumar et al., 2020; Oliveira et al., 2020; Rossini-Oliva et al., 2020). During last few decades, many methods have been developed to detect heavy metal ions in water, such as inductively coupled plasma atomic emission spectroscopy (ICP-AES), X-ray absorption spectroscopy (XAS), atomic absorption spectroscopy and so on. These methods have been widely applied in the laboratory and have excellent limits of detection and provide accurate quantifications. However, these methods do not meet the requirements for the simple and fast detection of metals in practical applications due to the complex sample preparation, high cost of instrumentation and complex analyses (Li et al., 2016; Bansod et al., 2017; Malik et al., 2019; Qi et al., 2020). Thus, an efficient, simple and sensitive method to detect and quantify heavy metal ions in aqueous media remains a challenge in environmental detection applications.

Carbon dots (CDs) are considered an ideal fluorescent material and have received significant attention as a result of their stable fluorescence, high optical absorptivity, good physicochemical stability, and biocompatibility; furthermore, they have been widely applied in medical diagnosis, bio-imaging, sensing, photocatalysis, optoelectronic devices and full-color displays (Sharma et al., 2017; Liu et al., 2019; Meng et al., 2019; Patel et al., 2019; Zhou et al., 2019). Recently, based on the fluorescence quenching effect caused by interactions between metal ions and the fluorophore functional groups, CDs have been demonstrated to be a promising fluorescent sensor for the detection of heavy metal ions, due to the unique dimensions and functional structure (Pang et al., 2017; Ansi and Renuka, 2019; Yarur et al., 2019; Yoo et al., 2019). Wang et al. (2015) used an acidic ionic liquid (SO_3_H-IL) as a catalyst and an ionic liquid ([Bmim]Cl) as a solvent to prepare CDs from microcrystalline cellulose. The CDs exhibited excellent, selective detection of Hg^2+^ along with good water dispersibility and photostability. In the concentration range 6–80 μmol/L, it was demonstrated that the CDs were an effective fluorescent sensor for the selective quantification of Hg^2+^, exhibiting a good linear relationship. Wang et al. (2019) also reported a simple hydrothermal method to synthesize nitrogen-doped CDs using an ethanolamine ionic liquid (1-carboxyethyl-3-methyl imidazole chloride) gel as a precursor. Due to the strong interactions between the metal ions and the surface groups and nitrogen atoms of the CDs, the CDs displayed significant heavy metal ion quenching ability for Hg^2+^ and Cu^2+^, as well as sensitive detection properties. Wu et al. (2017) summarized the relationship between the fluorescence quenching effect and the precursors and methods used. They found that, the chemically doped element (O, N, S), particle size and solvatochromic effects effectively controlled not only the fluorescence performance and specific detection ability of the CDs, but also endowed the CDs with excellent heavy metal detection abilities. Their results may provide a theoretical basis from which to develop novel and improved fluorescence performance.

Although these studies (Sharma et al., 2017; Ansi and Renuka, 2019; Liu et al., 2019; Meng et al., 2019; Wang et al., 2019; Yarur et al., 2019; Zhou et al., 2019) have achieved significant progress in the development of biocompatible CDs for heavy metal ion detection, most require toxic precursors and reagents, and expensive or complex preparation methods, especially when modifying the functional groups of CDs. The simultaneous satisfaction of these requirements remains highly challenging. Therefore, the development of a simple and effective strategy to design biocompatible CDs with sensing properties specific to the detection of heavy metal ions is necessary.

**SCHEME 1:**
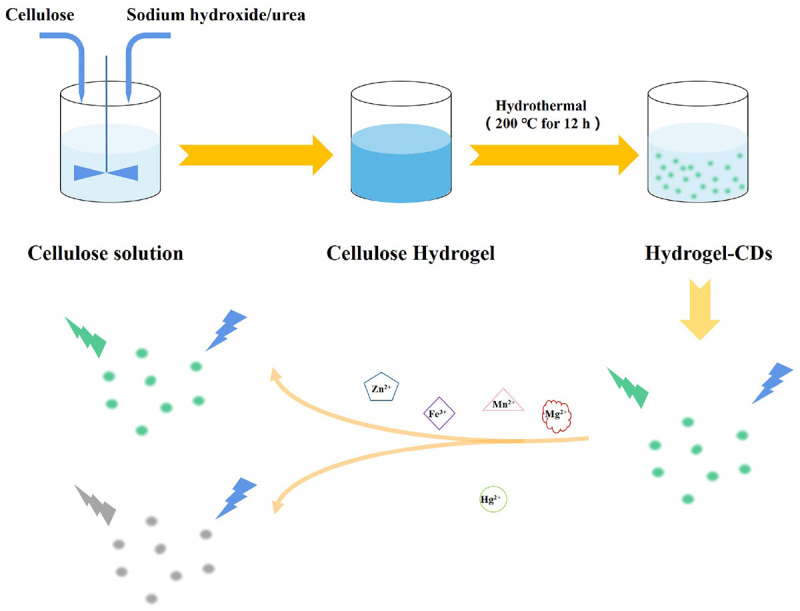
Synthetic procedure and mechanism of the sensing of CDs.

Herein, we report a simple strategy to develop biocompatible CDs based on a low-cost and natural polymer, cellulose hydrogel *via* a hydrothermal reaction. The prepared CDs-4a exhibited excellent fluorescence performance and good biocompatibility due to the small particle size and the functional groups on the particle surface, which could be used for labeling in living cells. Based on complexion between the metal ions and the functional groups of CDs-4a, the CDs-4a demonstrated the highly sensitive and specific detection of Hg^2+^ (Scheme 1). The CDs-4a maintained an excellent detection ability even in a complex solution containing various heavy metal ions.

## Materials and Methods

### Materials

Linter Cellulose (M_*w*_ = 8 × 10^4^) was provided by Hubei Chemical Fiber Group Co. Ltd. Epichlorohydrin (ECH), sodium hydroxide (NaOH), urea, copper sulfate (CuSO_4_), mercury nitrate (Hg(NO_3_)_2_), silver nitrate (AgNO_3_), manganese nitrate [Mn(NO_3_)_2_], chromium nitrate [Cr(NO_3_)_3_], lead nitrate [Pb(NO_3_)_2_], copper chloride (CuCl_2_), ferric chloride (FeCl_3_), calcium chloride (CaCl_2_), nickel chloride (NiCl_2_), cobalt chloride (CoCl_2_), zinc chloride (ZnCl_2_), and magnesium chloride (MgCl_2_) were purchased from Sinopharm Group, Shanghai, China. All reagents were of analytical reagent grade, and all chemicals were used without further purification.

### Preparation of CDs

Linter cellulose (4 g) was dissolved in a pre-cooled (−12°C) NaOH/urea aqueous solution (7/12 wt%, 100 g) to obtain the cellulose solution (Huang et al., 2016). Then, an amount of ECH (0.3 wt%) was added into the 4.5 g cellulose solution as crosslinking agent and stirred for 30 min at room temperature. Next, the homogeneous mixture was heated in the oven at 60°C for 4 h to obtain a uniform and transparent chemically crosslinked hydrogel. Finally, the hydrogel was soaked in deionized water for 48 h to completely remove the unreacted reagents from the pure cellulose hydrogel.

The pure cellulose hydrogel was placed in a hydrothermal reactor and heated at 200°C for 12 h. Then, the crude CDs formed were separated using high-speed centrifugation and stored in ethyl alcohol. Next, the purified CDs were separated using dialysis bags with different molecular weight cut-off values (10 and 1.2 k); they were named CDs-a (<1.2 k), CDs-b (1.2–10 k), CDs-c (>10 k), respectively. In order to investigate the factors influencing hydrogel content, the pure cellulose hydrogel was pretreated with different methods before the hydrothermal reaction as described in the following. (1) The pure cellulose hydrogel was used directly without pretreatment. (2) The pure 4.5 g cellulose hydrogels were immersed in a 7 wt% NaOH solution for 48 h. (3) The pure 4.5 g cellulose hydrogels were immersed in a 12 wt% aqueous urea solution for 48 h. (4) The pure 4.5 g cellulose hydrogels were immersed in a (7/12%, wt%) aqueous NaOH–urea solution for 48 h. The corresponding CDs were named as shown in Table 1.

**TABLE 1:** Samples name of CDs with different treatment.

	**Molecular weight cut-off (k)**		
**Pre-treatment**	**<1.2**	**1.2–10**	**>10**
CDs from pure hydrogel without pre-treatment	CDs-1a	CDs-1b	CDs-1c
CDs from NaOH pre-treated hydrogel	CDs-2a	CDs-2b	CDs-2c
CDs from urea pre-treated hydrogel	CDs-3a	CDs-3b	CDs-3c
CDs from NaOH/urea pre-treated hydrogel	CDs-4a	CDs-4b	CDs-4c

### Characterization

Fourier transform infrared (FTIR) spectra of the CDs were obtained using a Nicolet-Nexus 870 spectrophotometer. The UV/vis absorption of the CDs was measured using a CARY-100 UV/Vis spectrometer. The fluorescence spectra of the CDs were measured using a Hitachi F-4500. The morphology of the CDs was observed using a transmission electron microscope (TEM) at room temperature using a JEM-2010 (Rajendra et al., 2020). X-ray photoelectron spectroscopy (XPS) spectra were recorded with ESCALAB 250XI (Thermo Fisher Scientific, United States). The relative fluorescence quantum yield (QY) of the CDs was determined using methods previously reported in the literature (Shi et al., 2016; Khan et al., 2017; Liu et al., 2018). A quinine sulfate–H_2_SO_4_ solution (*n* = 1.33, QY = 0.54) was used as a reference solution.

The response of the CDs to metal stimuli was determined in a phosphate buffered saline (PBS) solution at pH 6.86. A 10 μmol/L aliquot of a solution containing different metal ions (Hg^2+^, Ag^+^, Mn^2+^, Cr^3+^, Ca^2+^, Fe^3+^, Ni^2+^, Cu^2+^, Co^2+^, Zn^2+^, Pb^2+^, Mg^2+^) was added into the CDs solution (50 μg/L). The fluorescence intensity at 450 nm was measured. Different metal ion solutions (10 μmol/L) were added into 3 mL of the CDs solution (50 μg/L), and the fluorescence intensity at emission wavelength of 450 nm was measured using fluorescence emission spectroscopy. Hg^2+^ solution with different concentration was added to the prepared CDs solution (50 μg/L), and the fluorescence intensity at emission wavelength of 450 nm was measured using fluorescence emission spectroscopy.

### *In vitro* Cytotoxicity Assay

The influence of CDs-4a on the proliferation properties of an FL cell line was determined using a cell counting kit-8 (CCK-8) assay. The FL cell line was seeded in 96-well culture plates with a density of 1 × 10^4^ cells/cm^2^ in a medium containing 10% fetal bovine serum and 1% penicillin–streptomycin. After 13 h of standard incubation, the cells adhered and spread over the bottom of the plate. Subsequently, the old cell culture medium was removed, and the cells were rinsed in the PBS solution. Next, 200 μL of fresh media with varying concentrations of CDs was added for the continuous cell culture. At intervals of 24, 48, and 72 h later, the CCK-8 assay was performed in a dark environment to quantify the number of living cells. Fluorescence images of the trypsin digests cells labeled using CDs-4a with different particle sizes were obtained at an absorbance of 450 nm using the microplate reader (Gao et al., 2018; Malina et al., 2019; Ren et al., 2019).

## Results and Discussion

### Preparation and Characterization of CDs

**FIGURE 1:**
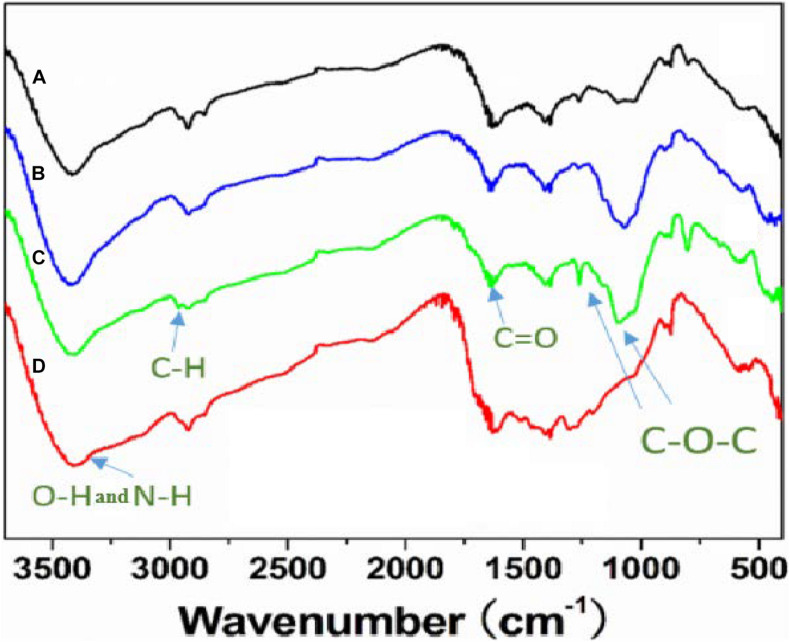
FTIR spectra of different CDs samples prepared from differently pre-treated cellulose hydrogels: **(A)** CDs-4a; **(B)** CDs-3a; **(C)** CDs-2a; **(D)** Cellulose.

**FIGURE 2:**
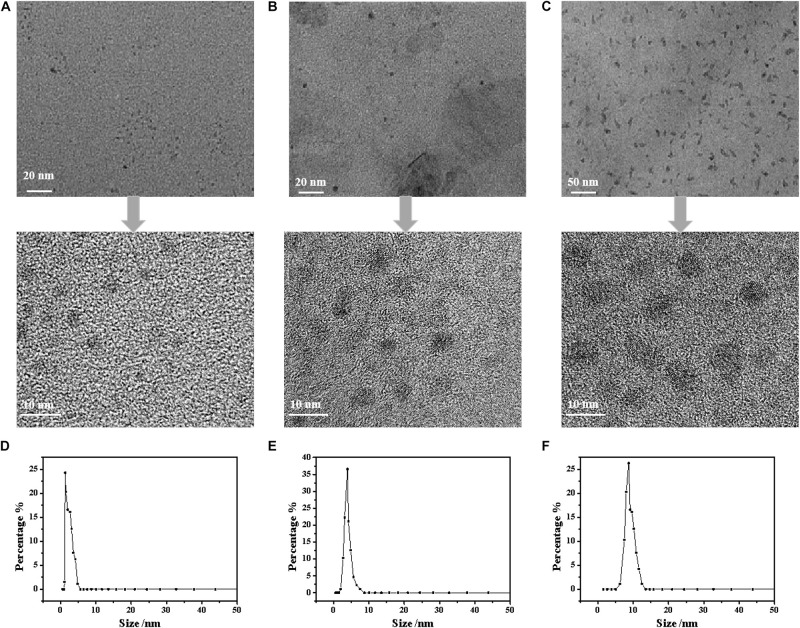
The TEM images of CDs-4: **(A)** CDs-4a; **(B)** CDs-4b, and **(C)** CDs-4c. And the particle size of CDs-4: **(D)** CDs-4a; **(E)** CDs-4b, and **(F)** CDs-4c.

The FTIR spectra of the CDs are shown in Figure 1. The characteristic peak of the CDs at 3450 cm^–1^ relates to the stretching vibration of –OH and –NH. The peak at 2910 cm^–1^ is the stretching vibration of –CH_3_. The characteristic peak at 1680 cm^–1^ corresponded to the stretching vibration of C=O, which resulted from C–OH oxidation during the hydrothermal reaction. This reaction is regarded as a luminescence emission source that can be used for fluorescence detection. It is worth noting that, compared with cellulose there was an obvious change of the characteristic peaks at 1230 cm^–1^ and 1100 cm^–1^ corresponding to the stretching vibrations of C–O–C, in the other types of CDs. This can be attributed to the ring-opening reaction of cellulose and its subsequent oxidation in the presence of NaOH or urea. The morphology of the NaOH/urea/cellulose CDs-4 was studied using TEM. As shown in Figures 2A–C, the CDs-4 particles were almost spherical in structure and exhibited no agglomeration or decomposition. In order to investigate the effect of particle size, the CDs-4 particles were separated into CDs-4a, CDs-4b, and CDs-4c using a dialysis bag with different molecular weight cut-off values; the higher the molecular weight, the bigger the particle size. Figure 2D shows the distribution of diameters for CDs-4a, CDs-4b, and CDs-4c were 2.11, 3.94, and 8.72 nm, respectively (Table 1).

### Fluorescence Properties of CDs

**FIGURE 3:**
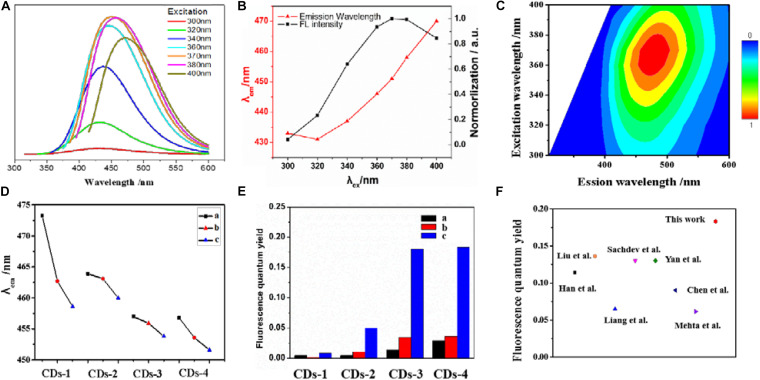
The excitation-dependent PL behavior of the CDs-4a aqueous solution **(A,B)**. **(C)** 2D excitation-emission contour map of CDs-4a at different excitation wavelengths. The emission wavelength of different CDs samples with the same excitation wavelength at 370 nm **(D)**. The fluorescence quantum yield of different CDs samples **(E)**. Comparison of fluorescence quantum yield of CDs-4a with reported CDs in the literatures **(F)**.

In order to investigate the fluorescence properties of CDs-4a, the excitation wavelength of CDs-4a was measured at wavelengths between 300 and 400 nm. As shown in Figures 3A–C, under the excitation wavelength of 370 nm, the fluorescence spectra of the CDs-4 exhibited a strong, single peak at 450 nm. Thus, an excitation wavelength of 370 nm was used for the following investigations. In addition, at the same excitation wavelength of 370 nm, it was found that the emission wavelength decreased as the particle size increased (Figure 3D). This results from the fact that the CDs particle size significantly affects the number of conjugated structures (Tao et al., 2017; Barman and Patra, 2018; Yan et al., 2019). Furthermore, the addition of NaOH or urea also affects the emission wavelength, leading to a blue shift. The maximum emission wavelengths of CDs-4a, CDs-4b, and CDs-4c were 450, 455, and 460 nm, respectively.

The relative fluorescence QY is an important factor in determining the fluorescence performance of fluorescent materials (Angshuman et al., 2016). Thus, the fluorescence QY of the different types of CDs was measured to evaluate the fluorescence properties. As shown in Figure 3E (Table 2), among all the types of CDs, CDs-1 exhibited the lowest QY. The introduction of NaOH or urea resulted in an obvious enhancement of the QY. Among all the samples, the CDs-4 exhibited the highest QY and the best fluorescence performance. Compared with most previously reported CDs, the QY of CDs-4a exhibits a higher performance of 0.183 (Figure 3F; Mehta et al., 2014; Sachdev and Gopinath, 2015; Chen et al., 2016; Liang et al., 2016; Yan et al., 2016; Liu et al., 2017; Han et al., 2018). This was a result of the effective promotion of the ring-opening and subsequent oxidation reaction of cellulose following the addition of NaOH or urea, which also provided increased doping of elements.

**TABLE 2:** The fluorescence quantum yield of different CDs samples.

**Sample**	**a**	**b**	**c**
CDs-1	0.008	0.002	0.005
CDs-2	0.050	0.011	0.005
CDs-3	0.179	0.034	0.013
CDs-4	0.183	0.036	0.029

**FIGURE 4:**
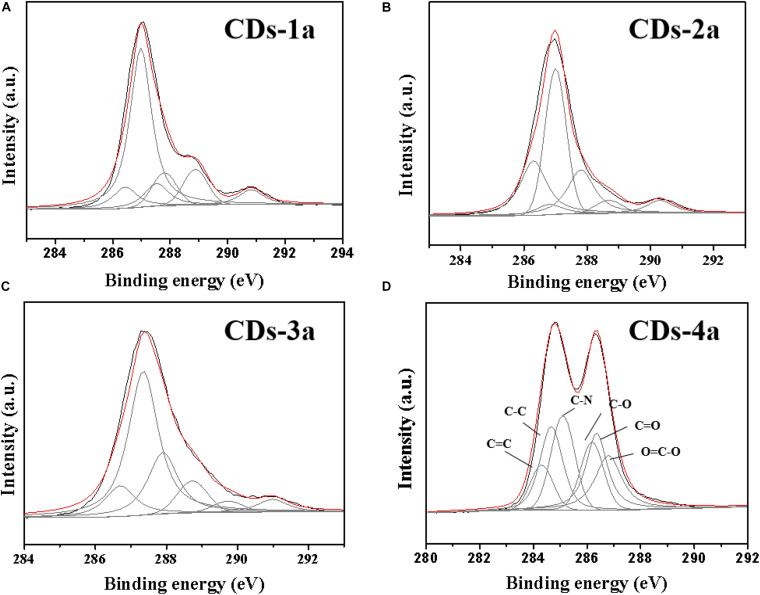
The XPS spectra of CDs: **(A)** CDs-1a; **(B)** CDs-2a; **(C)** CDs-3a, and **(D)** CDs-4a.

An XPS analysis of the CDs was performed (Figure 4 and Table 3). It was found that, CDs-3a and CDs-4a contained a higher nitrogen content than that of CDs-1a and CDs-2a. This demonstrated that, the addition of urea increased the amount of nitrogen functional groups in the CDs during the hydrothermal reaction. Furthermore, the C1S spectrum indicated that the amount of C=C/C=O and C–N functional groups in CDs-3a and CDs-4a was substantially higher than that of CDs-1a and CDs-2a, and as such an effective auxochrome was produced that enhanced the relative fluorescence emission efficiency. Hence, in comparison with the other samples, the CDs-3a and CDs-4a exhibited the best relative fluorescence emission efficiency.

**TABLE 3:** The elements content and functional groups of different CDs samples.

**Sample**	**C at%**	**N at%**	**O at%**	**C=C%**	**C=O%**	**C–N%**
CDs-1a	82.74	1.06	16.19	8.69	15.92	6.76
CDs-2a	86.07	1.59	12.35	9.13	16.46	3.05
CDs-3a	74.31	6.97	18.72	12.29	20.99	17.63
CDs-4a	74.15	7.77	18.07	14.62	21.05	19.18

In addition, for CDs pretreated using the same method, the particle size significantly influenced the QY. With increasing particle size, the QY obviously decreased. For example, the QY of CDs-4a was 0.18, which was much higher than that of CDs-4b and CDs-4c. This may be attributed to the smaller particle size and corresponding high number of luminous groups on the surface. Due to their excellent relative fluorescence emission efficiency, the CDs-4a were regarded as the ideal candidate for use in the remainder of this study.

### Biocompatibility of CDs-4

**FIGURE 5:**
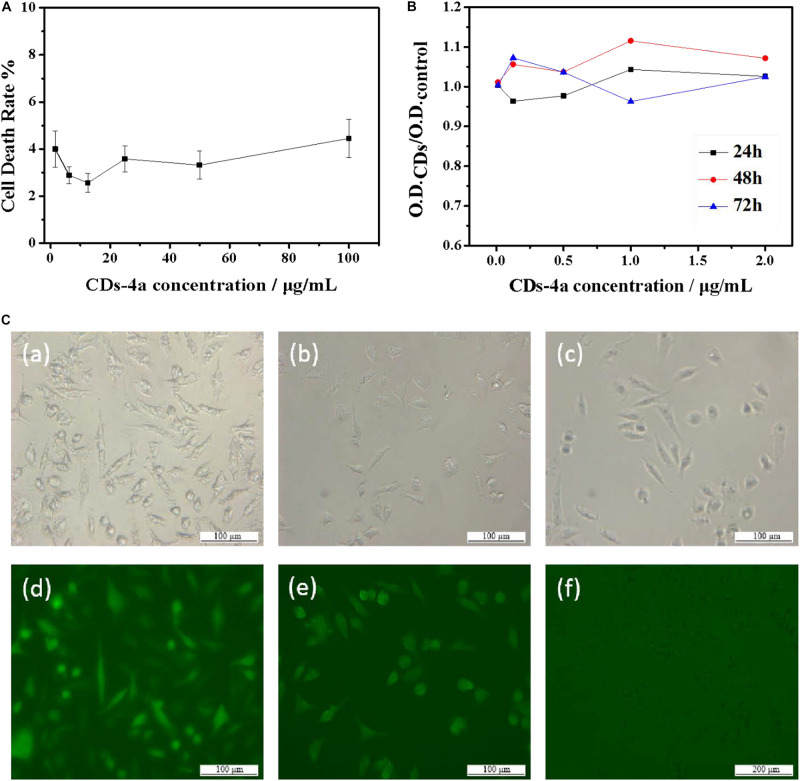
**(A)** Curve of acute toxicity test of CDs-4a with different concentration. **(B)** Cytostasis curve of CDs-4a with different concentration in 24, 48, and 72 h. **(C)** The FL cells images of **(a)** CDs-4a, **(b)** CDs-4b, **(c)** CDs-4c under visible light and **(d)** CDs-4a, **(e)** CDs-4b, **(f)** CDs-4c under the UV excitation.

In terms of future applications, assessment of the potential cytotoxicity of the biocompatible CDs is extremely important. As such, the biocompatibility of the CDs-4a was evaluated using FL cells. As shown in Figure 5A, after 12 h incubation, the cell death rate remained below 5%, even at high CDs-4a concentrations of up to 100 μg/mL. Cytostatic curves of the CDs-4a were obtained at different concentrations. Figure 5B shows the cell viability following treatment with different concentrations of CDs for 24, 48, and 72 h. The exert concentration of CDs at different concentrations did not result in a significant decrease in cell viability after 72 h, which suggests good biocompatibility. This result is similar with the results of other biocompatible CDs from natural polymer biomaterials (Sharma et al., 2017; Zhou et al., 2017; Zhang et al., 2018; Meng et al., 2019; Zulfajri et al., 2020).

Figure 5C shows fluorescence images of trypsin digests cells labeled using different sized CDs-4. The brightest regions are the CDs-4a, indicating they can be clearly distinguished; therefore, the CDs-4a displayed the best fluorescence performance. As well as a high QY value, the CDs-4a have a small particle size meaning they are easily taken up by cells. These cytotoxicity results imply that the CDs-4a prepared from hydrogels have potential for use in the labeling of living cells.

### Specific Detection of CDs-4a for Hg^2+^

**FIGURE 6:**
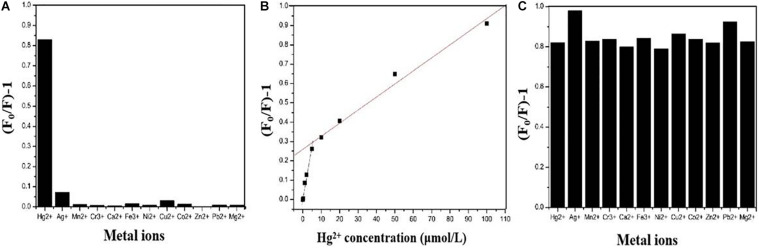
Fluorescent response of CDs-4a in the presence of 10 μM of different metal ions **(A)**. Plots of the values of (F_0_/F)-1 versus the concentration of Hg^2+^ **(B)**. Fluorescence response of CDs-4a in the presence of 10 μM Hg^2+^ and 10 μM another kind of metal ions **(C)**.

Figure 6A shows the fluorescence response performance of CDs-4a to different heavy metal ions. Compared with other metal ions, CDs-4a exhibited a specific response to Hg^2+^ ions. The addition of Hg^2+^ ions significantly quenched the fluorescence of CDs-4a, indicating promise for application in the selective detection of heavy metal ions. It could be attributed to the strong interactions between Hg^2+^ and functional groups of C=C/C=O and C–N of CDs-4a, which could effectively reduce the relative fluorescence strength. In order to evaluate the ability of the CDs-4a to detect Hg^2+^, the fluorescence quenching intensity of the CDs-4a was measured at various Hg^2+^ ion concentrations and calculated using the Stern-Volmer equation:

(1) $$\frac{F_{0}}{F}-1=\text{K}\,\left[Q\right]$$

Where *F*_0_ and *F* represent the fluorescence intensity of the fluorophore in the absence and presence of metal ions, respectively. K is the Stern-Volmer constant, and [*Q*] is the metal ion concentration. As shown in Figure 6B, the fluorescence quenching intensity obviously increased when the metal ion concentration was increased from 0.2 to 100 μmol/L. A two-stage, concentration-dependent fluorescence response was observed. Both stages exhibited a good linear relationship between (*F*_0_/*F* - 1) and concentration, ranging from 0.2 to 10 μmol/L (*R*^2^ = 0.9948, *K* = 5.88 × 10^4^) and from 10 to 100 μmol/L (*R*^2^ = 0.9911, *K* = 6.66 × 10^3^). The Stern-Volmer constant varies above and below a concentration of 10 μmol/L. In the low concentration stage, the slope is steep, and in the high concentration stage, the slope is less steep, which means that CDs-4a is more sensitive under lower concentration conditions. When the metal ion concentration was 0.2 μmol/L, the CDs-4a retained their Hg^2+^ detection ability and the fluorescence quenching intensity was maintain at approximately 0.1, which suggests a good sensitivity toward Hg^2+^ ions. Compared with most reports of Hg^2+^ ion detection, the CDs-4a displayed an excellent ability to detect Hg^2+^ ions (Table 4; Li et al., 2012; Wang et al., 2013; Desai et al., 2018; Xu et al., 2018; Zhang C. et al., 2020; Zhang H. et al., 2020). Based on the good linear relationship and low detection limit, the CDs-4a are able to specifically detect Hg^2+^ ions as well as quantify the concentration of Hg^2+^ ions.

**TABLE 4:** Detection limit and linear range of Hg^2+^ detectors.

**Detection probes**	**Linear range (μ M)**	**Detection limit (μ M)**	**References**
Hydrogel CDs	0.2–10, 10-100	0.2	This work
Eu^3+^-CDs	5–250	2.2	Desai et al., 2018
N,S-CDs	1–75	0.5	Xu et al., 2018
N-CQDs	0–25	0.23	Zhang H. et al., 2020
TFIC MNPs	4–16	5.04	Wang et al., 2013
Polymer sensor	1–30	0.728	Li et al., 2012
NCDs	0.9–10	0.15	Zhang C. et al., 2020

During practical applications, interference from other metal ions that co-exist with Hg^2+^ ions in solution may affect the detection. To study the effect of these interfering ions, further experiments were performed. The ability of CDs-4a to detect Hg^2+^ ions in the presence of other, interfering metal ions was determined. Figure 6C shows the fluorescence quenching intensity measured in a solution of Hg^2+^ mixed with other ions, it was observed that the fluorescence quenching intensity of almost all the other ions was similar to that of the pure Hg^2+^ solution, only Ag^+^ and Pb^2+^ slightly increased the fluorescence quenching intensity. These results indicate the CDs-4a have the capacity to resist interference from other ions during the detection of Hg^2+^ ions, which is significant in practical applications.

## Conclusion

In this work, a series of novel, biocompatible CDs were prepared from the natural polymer, cellulose hydrogel. Using an effective pretreatment method, the fluorescence performance was enhanced. Using dialysis bags with different molecular weight cut-off values, the CDs were separated into samples with different particle size. With the decrease of the particle size, the fluorescence QY of CDs increased. The CDs-4a exhibited good fluorescence during living cell labeling due to their small particle size and the presence of functional groups on the particle surface. More importantly, the CDs-4a exhibited specific detection of the heavy metal ion Hg^2+^ with high sensitivity. The LOD was as low as 0.2 μM and a two stage linear detection (0.2–10 and 10–100 μM) was observed. The CDs-4a also maintained an excellent detection ability, even in complex solutions containing interference metal ions, such as Fe^3+^, Zn^2+^, Mg^2+^, and Ni^2+^. We believe this work will provide novel insights into the development of high-performance multifunctional CDs based on a low-cost and natural polymer, cellulose hydrogel.

## Data Availability Statement

The original contributions presented in the study are included in the article/supplementary material, further inquiries can be directed to the corresponding authors.

## Author Contributions

MX and XW proposed the idea. HH and HG did the experiments. ZH helped the property evaluation of CDs. ZR helped the vitro cytotoxicity assay of CDs. All authors contributed to the article and approved the submitted version.

## Conflict of Interest

The authors declare that the research was conducted in the absence of any commercial or financial relationships that could be construed as a potential conflict of interest.
